# Fatal infantile mitochondrial encephalomyopathy, hypertrophic cardiomyopathy and optic atrophy associated with a homozygous *OPA1* mutation

**DOI:** 10.1136/jmedgenet-2015-103361

**Published:** 2015-11-11

**Authors:** Ronen Spiegel, Ann Saada, Padraig J Flannery, Florence Burté, Devorah Soiferman, Morad Khayat, Verónica Eisner, Eugene Vladovski, Robert W Taylor, Laurence A Bindoff, Avraham Shaag, Hanna Mandel, Ora Schuler-Furman, Stavit A Shalev, Orly Elpeleg, Patrick Yu-Wai-Man

**Affiliations:** 1 Pediatric Department B’, Genetic Institute, Emek Medical Center, Afula, Israel; 2 Genetic Institute, Emek Medical Center, Rappaport School of Medicine, Technion, Haifa, Israel; 3 Monique and Jacques Roboh Department of Genetic Research, Hebrew University, Hadassah Medical Center, Jerusalem, Israel; 4 Wellcome Trust Centre for Mitochondrial Research, Newcastle University, Newcastle upon Tyne, UK; 5 Department of Cellular and Molecular Biology, School of Biological Sciences, Pontificia Universidad Católica de Chile, Santiago, Chile; 6 Department of Pathology, Rambam Medical Center, Haifa, Israel; 7 Department of Clinical Medicine, University of Bergen, Bergen, Norway; 8 Metabolic Unit, Rambam Medical Center; 9 Department of Microbiology and Molecular Genetics, Hebrew University, Hadassah Medical Center, Jerusalem, Israel; 10 Newcastle Eye Centre, Royal Victoria Infirmary, Newcastle upon Tyne, UK

**Keywords:** Clinical genetics, Metabolic disorders, Neuroophthalmology, Neuromuscular disease

## Abstract

**Background:**

Infantile-onset encephalopathy and hypertrophic cardiomyopathy caused by mitochondrial oxidative phosphorylation defects are genetically heterogeneous with defects involving both the mitochondrial and nuclear genomes.

**Objective:**

To identify the causative genetic defect in two sisters presenting with lethal infantile encephalopathy, hypertrophic cardiomyopathy and optic atrophy.

**Methods:**

We describe a comprehensive clinical, biochemical and molecular genetic investigation of two affected siblings from a consanguineous family. Molecular genetic analysis was done by a combined approach involving genome-wide autozygosity mapping and next-generation exome sequencing. Biochemical analysis was done by enzymatic analysis and Western blot. Evidence for mitochondrial DNA (mtDNA) instability was investigated using long-range and real-time PCR assays. Mitochondrial cristae morphology was assessed with transmission electron microscopy.

**Results:**

Both affected sisters presented with a similar cluster of neurodevelopmental deficits marked by failure to thrive, generalised neuromuscular weakness and optic atrophy. The disease progression was ultimately fatal with severe encephalopathy and hypertrophic cardiomyopathy. Mitochondrial respiratory chain complex activities were globally decreased in skeletal muscle biopsies. They were found to be homozygous for a novel c.1601T>G (p.Leu534Arg) mutation in the *OPA1* gene, which resulted in a marked loss of steady-state levels of the native OPA1 protein. We observed severe mtDNA depletion in DNA extracted from the patients’ muscle biopsies. Mitochondrial morphology was consistent with abnormal mitochondrial membrane fusion.

**Conclusions:**

We have established, for the first time, a causal link between a pathogenic homozygous *OPA1* mutation and human disease. The fatal multisystemic manifestations observed further extend the complex phenotype associated with pathogenic *OPA1* mutations, in particular the previously unreported association with hypertrophic cardiomyopathy. Our findings further emphasise the vital role played by OPA1 in mitochondrial biogenesis and mtDNA maintenance.

Mitochondrial disorders are genetically and phenotypically highly heterogeneous. Due to the limited coding capacity of the mitochondrial genome, the majority of structural and accessory proteins required for the proper assembly and functioning of the mitochondrial respiratory chain are encoded by the nuclear genome. Mutations in an increasing number of these critical nuclear-encoded genes have been identified over the past decade by next-generation sequencing. These advances have allowed the elucidation of the underlying genetic diagnosis in patients with complex phenotypes and also broadened the spectrum of clinical manifestations associated with specific disease-causing genes. Syndromes characterised by infantile-onset hypertrophic cardiomyopathy and encephalopathy are genetically heterogeneous, but mitochondrial dysfunction with impairment of mitochondrial oxidative phosphorylation has emerged as an important pathophysiological mechanism. Accordingly, these specific syndromes may be caused by mutations in various genes involving both the mitochondrial or nuclear genomes. Unsurprisingly, a number of nuclear genes causing isolated or combined mitochondrial respiratory chain defects have been identified in patients with infantile hypertrophic cardiomyopathy and variable forms of encephalopathy, of which recent examples include *NDUFV2*, *COX6B1*, and *SCO2*.^1–3^

Heterozygous *OPA1* mutations cause autosomal dominant optic atrophy (DOA), which is the most common form of inherited mitochondrial blindness with a minimum prevalence of 1 in 25 000 in the general population.^4^ Here, we report on two siblings from a consanguineous family who presented with a fatal, infantile-onset, global encephalopathy and progressive hypertrophic cardiomyopathy due to a novel homozygous mutation in the *OPA1* gene. Homozygous *OPA1* mutations have not been reported previously and the unexpected association with cardiac involvement further broadens the genotypic and phenotypic spectrum associated with syndromic DOA.

The subjects of this study are two sisters of Arab Muslim origin and their parents are entirely healthy first-degree cousins with no visual or neurological complaints. Patient I-1 was born prematurely at 35 weeks gestation and failure to thrive was a problem since birth. At 2 months of age, she was also noted to have truncal hypotonia that was replaced by multiple episodes of hypertonia and opisthotonic posturing. Furthermore, she had a weak cry and abnormal eye pursuits. An initial echocardiography and ophthalmological examinations performed at that time were unremarkable. Metabolic investigations revealed elevated serum alanine and lactate levels, but a normal cerebrospinal fluid (CSF) lactate concentration. Disease progression was marked by a persisting failure to thrive with severe neurodevelopmental delay, but brain MRI was normal. The patient subsequently developed a progressive, non-obstructive, generalised hypertrophic cardiomyopathy, in the absence of overt heart failure. The patient died at 10 months of age following an apnoeic episode.

Patient I-2 was born at term following a caesarean section due to breech presentation. Two days later, she developed generalised hypertonia and opisthotonic posturing, and feeding was inadequate due to poor suckling. Initial echocardiography showed mild thickening of the left ventricular myocardium. Serum and CSF lactate concentrations were both elevated. Brain MRI performed at 1.5 months of age was normal and no abnormal lactate peaks were detected on functional spectroscopic imaging. Due to gastro-oesophageal reflux and persisting failure to thrive, a gastrostomy tube and Nissen funduplication procedures were performed at 3 months of age. The disease course was further complicated by progressive, hypertrophic, non-obstructive cardiomyopathy, profound neurodevelopmental retardation, hypotonia with significant muscle wasting and sensorineural deafness. There was no nystagmus, ptosis or limitation of eye movements. Ophthalmological examination at 6 months of age revealed mild pallor of both optic nerves. Visual electrophysiology revealed severely attenuated visual evoked potentials, consistent with primary retinal ganglion cell pathology, and the electroretinogram responses were significantly reduced, indicative of more global retinal degeneration. The patient died at 11 months of age following an apnoeic episode while sleeping.

A muscle biopsy was performed at 8 months of age (patient I-1) and at 1 month of age (patient I-2). Routine mitochondrial immunohistochemical staining was apparently normal with no cytochrome *c* oxidase-deficient muscle fibres. Enzymatic activities of the five respiratory chain complexes and citrate synthase (CS) activity, a mitochondrial matrix enzyme marker, were determined in isolated muscle mitochondria by spectrophotometric assays. This analysis revealed global decrease in all the respiratory chain complexes when normalised to CS activity in both patients, with complexes I and IV being the most affected (table 1) and complex II relatively preserved.

**Table 1:** Enzymatic activities of mitochondrial respiratory chain complexes

Assay	Patient I-1	Patient I-2	Controls±SD (n=50)
Complex I	0.047 (24%)	0.050 (25%)	0.199±0.043
Complex I+III	0.087 (40%)	n.d.	0.217±0.599
Complex II	0.099 (64%)	0.091 (59%)	0.154±0.024
Complex II+III	0.100 (65%)	0.053 (34%)	0.153±0.039
Complex III	0.934 (46%)	n.d.	2.01±0.40
Complex IV	0.270 (21%)	0.230 (22%)	1.03±0.24
Complex V	0.151 (44%)	n.d.	0.34±0.096

*mU/U citrate synthase (% of control).

n.d. not determined.

Molecular genetic investigations for both sisters were initiated after obtaining the relevant written informed consents and local ethical review board approval. Because of parental consanguinity, genome-wide homozygosity mapping for both affected sisters was undertaken using Affymetrix Gene-Chip Human Mapping 50 K Xba Array and Gene-Chip Human Mapping 250 K NspI Array. We specifically looked for genes with proven or suspected mitochondrial function in light of the generalised oxidative phosphorylation defects detected in both siblings. We identified four large homozygous chromosomal stretches shared by both siblings (see online supplementary table S1), which harboured >150 genes, of which nine had putative mitochondrial involvement. Whole exome sequencing (HiSeq 2000, mean depth coverage of X 65.9; see online supplementary methods) and bioinformatic filtering highlighted a homozygous c.1601T>G variant in the *OPA1* gene, predicting the substitution of a highly evolutionary conserved leucine at position 534 with arginine (p.Leu534Arg, *OPA1* NM_015560.2; see online supplementary figure S1) as the only mitochondrial-targeted gene that could be functionally relevant. This previously unreported *OPA1* variant affects the GTPase catalytic domain (http://mitodyn.org/home.php?select_db=OPA1, accessed 22 June 2015) and it was predicted to be disease causing by Mutation Taster biotool (probability score 0.999, range 0–1.0) and SIFT (Sorting Intolerant from Tolerant, probability score 1.0, range, 0–1.0) prediction softwares. Both parents were heterozygous for this *OPA1* variant. They did not report any visual difficulties and a comprehensive neuro-ophthalmological evaluation was normal. Their optic discs did not look pale on dilated fundus examination and optical coherence tomography (OCT) imaging did not show any subclinical thinning of the retinal nerve fibre layer. The parents have two clinically unaffected sons who were 14 and 11 years old when last reviewed in clinic. The older son is heterozygous for the c.1601T>G *OPA1* variant, whereas the other son only carries the wild-type *OPA1* allele. This novel *OPA1* variant was not detected in a cohort of 120 control subjects of similar ethnic origin and it was not present in a database including 63 000 exome sequences (Exome Aggregation Consortium; http://exac.broadinstitute.org, accessed 22 June 2015).

In order to further assess the pathogenicity of this c.1601T>G *OPA1* variant, western blot analysis was performed on homogenate muscle tissue from patient I–1 using standard monoclonal antibodies that bind to the C-terminus region of the OPA1 protein. There was a significant reduction in steady-state OPA1 protein level in patient I–1 compared with age-matched controls (figure 1A,B). This observation was consistent with the reduced OPA1 protein levels observed in another patient (NO-1) with a complicated syndromic DOA phenotype characterised by optic atrophy and marked generalised neurodegenerative features, who has been confirmed to harbour compound heterozygous *OPA1* missense mutations in exon 5b (c.768C>G, p.Ser256Arg) and exon 8 (c.854A>G, p.Gln285Arg; figure 1C). To further investigate how a homozygous missense *OPA1* mutation could result in markedly reduced OPA1 protein level, in silico protein modelling was carried out. The c.1601T>G *OPA1* variant replaces a highly hydrophobic and evolutionary conserved leucine residue with an arginine residue. Our modelling work suggests that this amino acid substitution, in the close vicinity of the protein's critical GTPase domain, could destabilise the protein, accounting for the significantly reduced OPA1 protein level observed on western blot analysis (see online supplementary figure S2).

**Figure 1:**
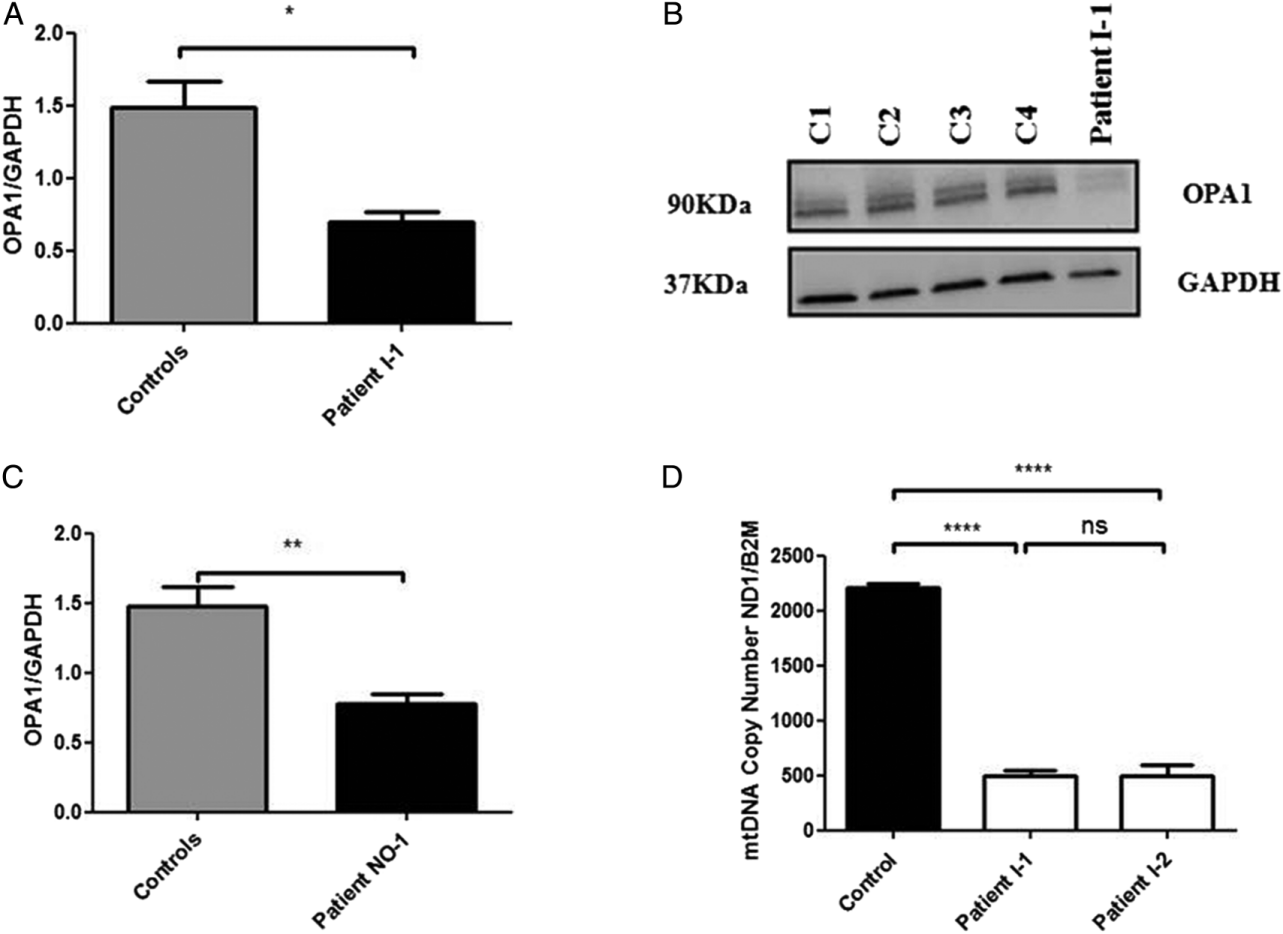
(A) Densitometric analysis of OPA1 in muscle tissue from patient I-1. The level of the OPA1 protein was normalised against GAPDH. Four different control muscle samples were used. Error bars represent SE of the mean. An unpaired Student's t test was used in statistical comparison between patient and controls (p=0.0144). (B) Western blot illustrating OPA1 protein levels in muscle controls and muscle tissue of patient I-1. (C) Densitometric analysis of OPA1 in fibroblasts from patient NO-1 who carries compound heterozygous, c.768C>G (p.Ser256Arg in exon 5b) and c.854A>G (p.Gln285Arg in exon 8).^8^ The level of OPA1 protein was normalised against GAPDH. Four different control muscle samples were used. An unpaired Student's t test was used in statistical comparison between patient and controls (p=0.0039). (D) MtDNA copy number in skeletal muscle from the two affected sisters compared with controls. The analysis was performed using a SYBR Green real-time PCR assay with the mtDNA gene *MTND1* corrected against the nuclear gene *B2M* to calculate mtDNA copy number. Error bars represent SE of the mean. Statistical analysis performed with Student's unpaired t test (p<0.0001). *Level of statistical significance.

Long-range PCR performed on homogenate DNA extracted from the muscle biopsy specimens of the two affected sisters did not reveal any multiple mitochondrial DNA (mtDNA) deletions (see online supplementary figure S3). However, there was evidence of significant mtDNA depletion with a 78% decrease for patient I-1 and a 78% decrease for patient I-2 compared with control values (figure 1D). Transmission electron microscopy (TEM) assay of muscle biopsy from patient I-2 disclosed large mitochondria with incomplete fusion of the inner mitochondrial membrane (see online supplementary figure S4).

*OPA1* encodes for a large multimeric dynamin-like GTPase protein, which localises to the inner mitochondrial membrane where it plays a crucial role in mediating mitochondrial fusion.^4^ In addition, OPA1 is essential for the assembly and stability of the mitochondrial respiratory chain supercomplexes,^5^ mitochondrial cristae organisation,^6^ the sequestration of pro-apoptotic cytochrome *c* molecules within these tight junctions and, importantly, mtDNA maintenance.^7^ Heterozygous *OPA1* mutations account for the majority of cases of non-syndromic DOA, which classically presents in early childhood with progressive visual loss. Although optic atrophy is the defining feature of DOA, non-penetrance has been reported in 10%–20% of *OPA1* mutation carriers.^4^ Furthermore, several papers and recent reports highlighted the fact that a significant proportion of *OPA1* mutation carriers, up to 20% in a large case series, can develop a more severe syndromic form of the disease (termed DOA plus) where the visual loss is compounded by a cluster of neurological deficits that include chronic progressive external ophthalmoplegia, sensorineural hearing loss, ataxia, myopathy and peripheral neuropathy.^8^ Interestingly, the extraocular manifestations of pathogenic *OPA1* mutations can sometimes develop in the absence of overt optic atrophy and visual failure.^9^ ^10^ As expected, bi-allelic *OPA1* mutations result in a more severe phenotype characterised by an earlier age of onset and with a more aggressive neurological course—referred to by some investigators as Behr syndrome.^10–12^ More recently, a deep intronic *OPA1* mutation was shown to behave as an intralocus modifier that results in DOA plus phenotypes when it occurs in conjunction with an exonic *OPA1* missense variant (c.1146A>G, p.Ile382Met; NM_015560.2)—the latter being insufficient to cause disease on its own.^13^ When present in the heterozygous state, the c.1601T>G *OPA1* variant in our family apparently does not result in clinical or subclinical disease. Taken together, these observation further add to the complexity of genotype–phenotype correlations and it demonstrates the spectrum of *OPA1* mutations from the classical dominant mutations (which accounts for the majority of cases), to compound heterozygous mutations causing more severe syndromes (including Behr syndrome), and our current report with a homozygous mutation.

Our case report provides a number of new insights into the molecular and phenotypic manifestations of pathogenic *OPA1* mutations. First, the two affected siblings harboured a novel homozygous *OPA1* variant, which have not been reported previously in association with human disease. These two patients developed a rapidly progressive lethal phenotype beginning in early infancy and characterised by marked multisystemic involvement due to generalised mitochondrial respiratory chain dysfunction. This ultimately fatal clinical presentation is consistent with the rather striking observations made in three *Opa1* mouse models. Homozygous mutant mice died in utero during embryogenesis due to severe abrogation of Opa1 protein levels, confirming a critical role for this pro-fusion mediator in early life and development.^14–16^ In silico modelling indicates that the c.1601T>G OPA1 missense mutation affects the integrity of the protein structure in the vicinity of the catalytic GTPase domain, providing a plausible explanation for the significant reduction in OPA1 level confirmed by western blot analysis. OPA1 also forms oligomeric complexes in vivo and the lack of any wild-type protein due to the homozygous mutations could further exacerbate protein stability due to impaired oligomerisation.

Carriers harbouring missense *OPA1* mutations affecting the catalytic GTPase region have a significantly increased risk of developing the more severe DOA plus phenotype,^8^ ^9^ which is again consistent with the rapidly fatal clinical progression observed for the two affected sisters. Second, hypertrophic cardiomyopathy has not been reported previously to be associated with human *OPA1* mutations. Interestingly, histopathological evidence of cardiomyopathy was apparent in a heterozygous mutant *Opa1* mouse model as early as at 5 months of age^16^ and the affected cardiac tissues exhibited a combined complex I and IV respiratory chain defect with reduced ATP production.^17^ Importantly, previous reports have noted cardiac manifestations in a number of patients with DOA plus including symptomatic tachycardia,^18^ early-onset myocardial infarction^19^ and sinus bradycardia.^20^ These potentially life-threatening associations are important and further investigations of a larger cohort of patients with DOA will clarify whether this patient group is at risk of clinical or subclinical cardiac involvement, and if so, the need for prophylactic screening and intervention in at-risk mutation carriers.

Although the precise disease mechanisms by which *OPA1* mutations lead to human disease is still under intense investigation, a growing body of evidence points towards a synergy between disturbed mitochondrial dynamics, mtDNA instability and impaired mitochondrial oxidative phosphorylation, which eventually precipitates an irreversible molecular cascade towards cell death. Several studies have demonstrated reduced activities of various mitochondrial respiratory chain complexes, in particular complex I and complex IV, in muscle biopsies and fibroblasts obtained from patients with DOA and confirmed *OPA1* mutations.^8^ ^21^ ^22^ In keeping with these findings, muscle biopsies from both affected sisters showed a marked global deficiency in mitochondrial respiratory chain activity, which was relatively more pronounced for complexes I and IV. It is therefore biologically plausible that such a severe impairment in mitochondrial oxidative phosphorylation would have a catastrophic consequence for tissues with a high basal level of energy consumption such as skeletal muscle, cardiac muscle and the central nervous system, in addition to the retina.

In addition to the generalised cellular energy shortage, OPA1 is now well established as a regulatory factor involved in mtDNA maintenance and genome stability. Previous studies have clearly shown that mtDNA deletions can accumulate to high levels in postmitotic tissues of affected *OPA1* mutation carriers.^9^ ^22^ MtDNA deletions were not detected in the two affected sisters, which is not surprising given their very young age and the need for clonal expansion of somatic mtDNA abnormalities to proceed over several decades before they become detectable using routine PCR-based detection techniques. However, mtDNA depletion was clearly demonstrated in both patients—a striking finding that has not been reported before with pathogenic *OPA1* mutations. This observation is mechanistically important and support a number of recent studies linking OPA1 and also its sister protein mitofusin 2 (MFN2), with the complex process of mtDNA replication. OPA1 is thought to physically anchor nucleoids to the inner mitochondrial membrane via the peptide segment encoded by exon 4b, and using an elegant series of mouse models, marked mtDNA depletion was observed in the context *OPA1* null cells.^7^ ^23^ MFN2 is a major pro-fusion located within the outer mitochondrial membrane and it works in tandem with OPA1 to control the sequential steps that eventually lead to mitochondrial fusion. Supporting evidence for abnormal inner mitochondrial membrane fusion was provided by TEM of patient’s I-2 muscle biopsy. As for *OPA1* mutations, *MFN2* mutations predominantly cause the accumulation of multiple mtDNA deletions, but a recent case report indicates that mtDNA depletion can occur as well.^24^

It is now abundantly clear that the deleterious consequences of pathogenic *OPA1* mutations are not limited to the optic nerve. We have further extended the mutational and phenotypic spectrum of *OPA1* disease to include lethal infantile mitochondrial encephalomyopathy and hypertrophic cardiomyopathy secondary to a novel homozygous missense mutation that targets the key functional GTPase domain. Our case report also further reinforces the power of next-generation exome sequencing in elucidating the genetic aetiology of complex disease phenotypes when combined with rigorous phenotyping and a clear a priori list of candidate genes, in this particular family, *OPA1* given the clear link with optic atrophy.

## Supplementary Material

Web figures

Web method

Web table
