# Supplementary material for: Fatal infantile mitochondrial encephalomyopathy, hypertrophic cardiomyopathy and optic atrophy associated with a homozygous OPA1 mutation
Source: J Med Genet. 2015 Nov 11;53(2):127–31. doi: 10.1136/jmedgenet-2015-103361 (PMC4752660; doi:10.1136/jmedgenet-2015-103361)
Supplement: Web figures [file jmedgenet-2015-103361-s1.pdf]

Supplementary Figure 1

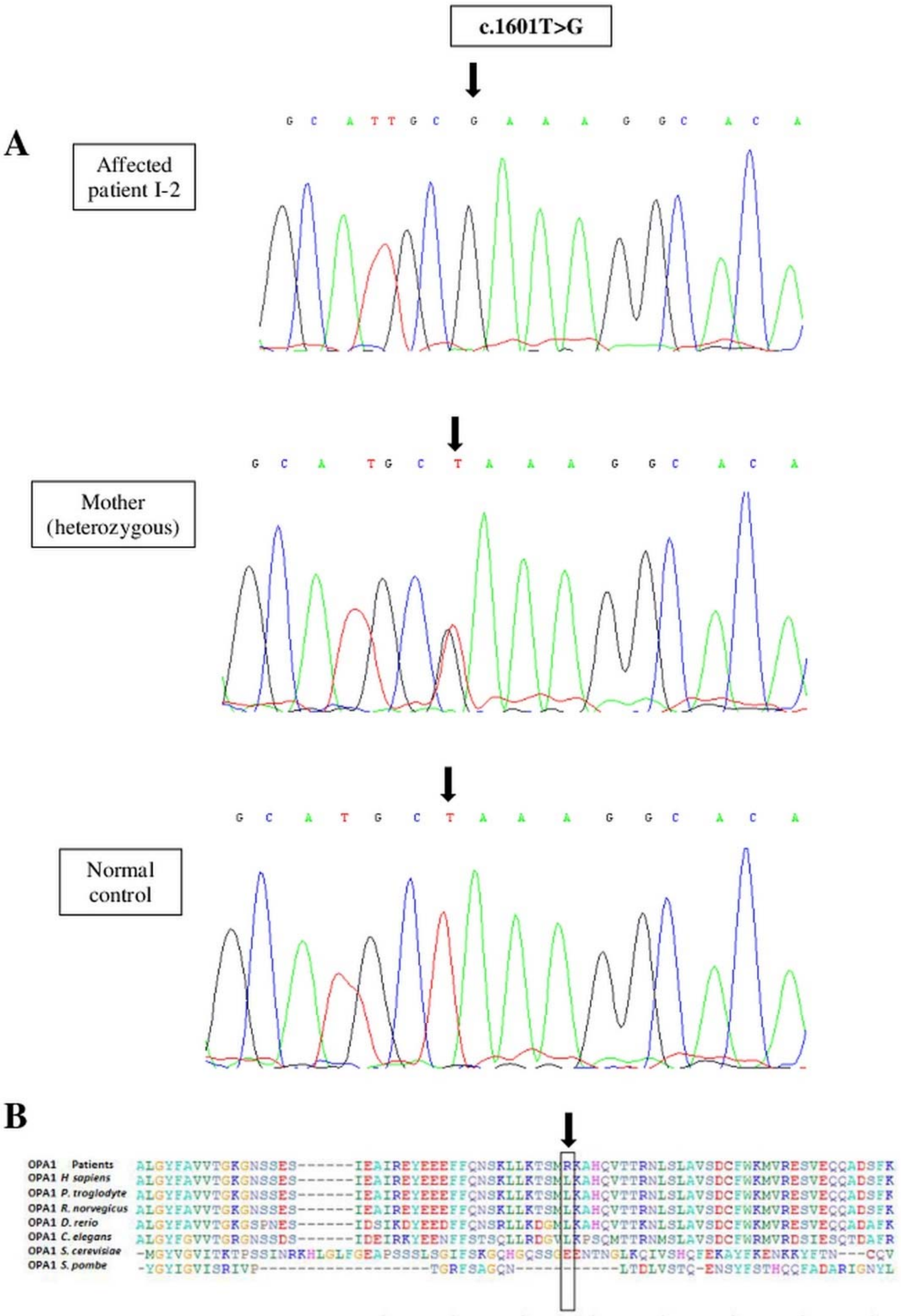

Supplementary Figure 2

Supplementary Figure 4:

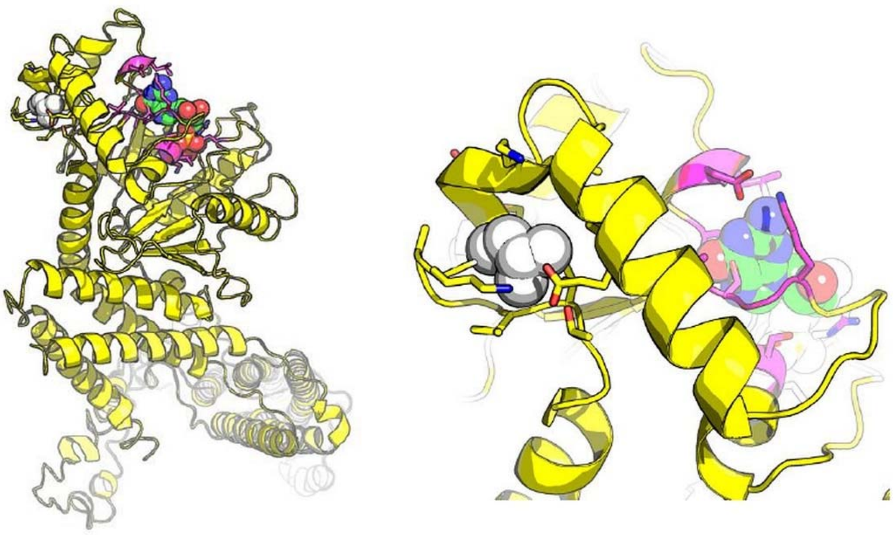

Supplementary Figure 3

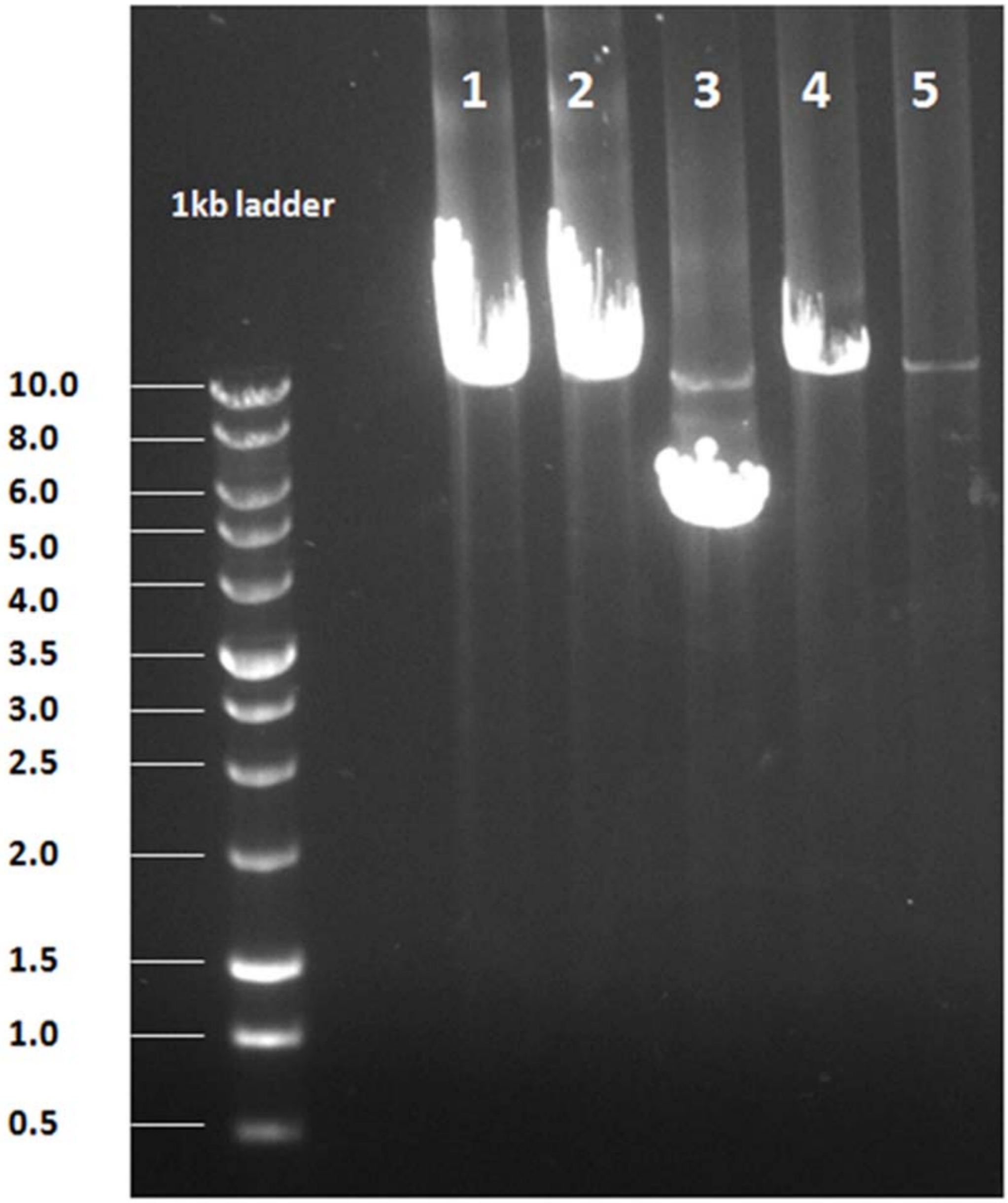

Supplementary Figure 4

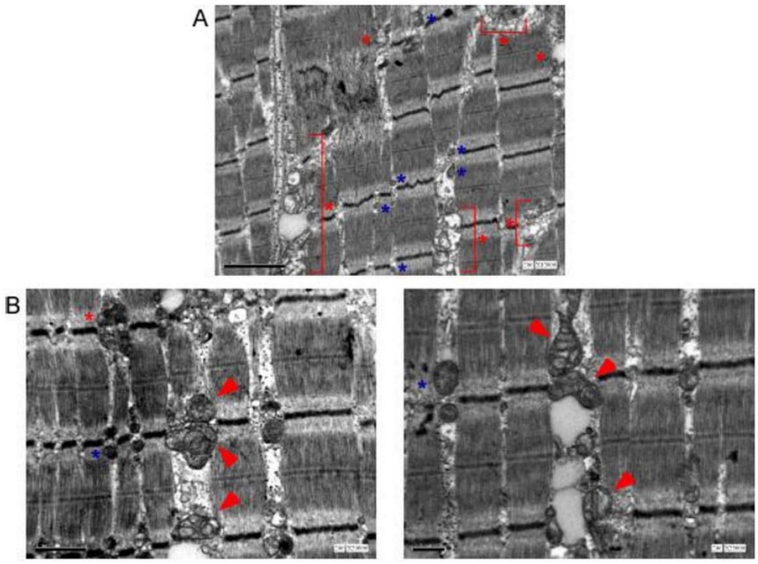

* Apparently normal structure

* Mitochondria with aberrant ultrastructure

► Aberrant mitochondria, showing details of clustered matrix-carring-IMM, surrounded by OMM
