# Supplementary material for: Fatal infantile mitochondrial encephalomyopathy, hypertrophic cardiomyopathy and optic atrophy associated with a homozygous OPA1 mutation
Source: J Med Genet. 2015 Nov 11;53(2):127–31. doi: 10.1136/jmedgenet-2015-103361 (PMC4752660; doi:10.1136/jmedgenet-2015-103361)
Supplement: Web method [file jmedgenet-2015-103361-s2.pdf]

The resultant 56.94 million reads were aligned, by the DNAnexus software (Palo Alto,CA, USA) with the human genome assembly hg19 (GRCh37) as reference. Of the called variants, only ten were homozygous, at a minimal depth of X6, on-target, non- synonymous, and with a MAF<0.1% at dbSNP138 and MAF<1% in the Hadassah in-house dbSNP (table).

chr	nt (Hg19)	ref	mut	known SNP	gene	AA change
1	230914853	C	T	rs148341318	CAPN9	T-363-M
1	235331875	C	T		ARID4B	A-1302-T
3	193364865	T	G		OPA1	L-535-R
3	197880164	GCAGCA			FAM157A	QQQ-81-Q
9	90501759	G	T	rs80294513	SPATA31E1	R-786-L
15	79603608	C	T	rs370013859	TMED3	P-6-L
15	89399986	C	G	rs201822759	ACAN	D-1390-E
17	38519452	C	A		GJD3	V-206-L
19	37487873	C	T	rs1667364	ZNF568	A-427-V
19	58967064	C	A		ZNF324B	H-251-Q
