# Supplementary material for: Fatal infantile mitochondrial encephalomyopathy, hypertrophic cardiomyopathy and optic atrophy associated with a homozygous OPA1 mutation
Source: J Med Genet. 2015 Nov 11;53(2):127–31. doi: 10.1136/jmedgenet-2015-103361 (PMC4752660; doi:10.1136/jmedgenet-2015-103361)
Supplement: Web table [file jmedgenet-2015-103361-s3.pdf]

Supplementary Table 1: Homozygous regions shared by the two affected siblings.

Chromosome	Start Position	End Position
3	190090661	194028647
4	127448140	129685158
5	44626810	50328257
8	77570266	78463539

The start and end point coordinates are in relation to the human genome reference sequence hg19.
